# Bayesian evidence synthesis as a flexible alternative to meta-analysis: A simulation study and empirical demonstration

**DOI:** 10.3758/s13428-024-02350-2

**Published:** 2024-03-26

**Authors:** Elise van Wonderen, Mariëlle Zondervan-Zwijnenburg, Irene Klugkist

**Affiliations:** 1 Amsterdam Center for Language and Communication, University of Amsterdam, Spuistraat 134, Amsterdam, 1012 VB The Netherlands; 2 Department of Methodology & Statistics, Utrecht University, Utrecht, The Netherlands

**Keywords:** Research synthesis, Bayes factor, Robustness, Conceptual replications, Experimental psychology, Informative hypotheses

## Abstract

Synthesizing results across multiple studies is a popular way to increase the robustness of scientific findings. The most well-known method for doing this is meta-analysis. However, because meta-analysis requires conceptually comparable effect sizes with the same statistical form, meta-analysis may not be possible when studies are highly diverse in terms of their research design, participant characteristics, or operationalization of key variables. In these situations, Bayesian evidence synthesis may constitute a flexible and feasible alternative, as this method combines studies at the hypothesis level rather than at the level of the effect size. This method therefore poses less constraints on the studies to be combined. In this study, we introduce Bayesian evidence synthesis and show through simulations when this method diverges from what would be expected in a meta-analysis to help researchers correctly interpret the synthesis results. As an empirical demonstration, we also apply Bayesian evidence synthesis to a published meta-analysis on statistical learning in people with and without developmental language disorder. We highlight the strengths and weaknesses of the proposed method and offer suggestions for future research.

## Introduction

Science is by nature a cumulative endeavor, in which we build upon results from previous studies to inform theory and generate new hypotheses. However, a great challenge in building theories is the high prevalence of conflicting results in psychological research, caused in part by small sample sizes and reliance on null hypothesis significance testing (e.g., Button et al., 2013; Open Science Collaboration, 2015; Van Calster et al., 2018). A commonly used method to help remedy this issue is to increase the robustness of scientific findings by means of meta-analysis (e.g., Cooper et al., 2019; Lipsey & Wilson, 2001). In a meta-analysis, the researcher quantitatively summarizes results across studies by computing a weighted mean effect size and corresponding confidence intervals to investigate whether there is evidence for an effect when all studies are taken together. Meta-analysis therefore helps mitigate the issue of underpowered studies, as a small effect may be statistically non-significant in each individual study, but significant when all these studies are combined. Furthermore, it can be investigated whether there is significant heterogeneity in effect sizes across studies, and if so, which study-level variables can explain part of this variation (e.g., Berkey et al., 1995; van Houwelingen et al., 2002). This way, meta-analysis can help explain conflicting results in the literature and contribute to more coherent theories.

Although meta-analysis is a powerful and versatile method for aggregating multiple studies, conducting a meta-analysis is sometimes challenging or even impossible for the set of studies a researcher wishes to combine. As studies are combined at the level of the effect size, meta-analysis requires the effect sizes across studies to be conceptually comparable and have the same statistical form (Lipsey & Wilson, 2001). These requirements are unlikely to be met if studies differ considerably regarding research design, operationalization of key variables, and statistical models used. For example, when studies have measured variables on different scales (e.g., continuous vs. binary), transformations are needed to translate the effect sizes into one common effect-size metric. Although such transformations exist (see, e.g., Cooper et al., 2019; Lipsey & Wilson, 2001), they do not exist between all effect-size metrics and many of these transformations “make strong or even untenable assumptions” (van Assen et al., 2022, p. 1), begging the question of whether effect sizes that require such transformations should be combined in the first place. Furthermore, even small differences in design often result in different population effects being estimated, which means their effect sizes cannot be directly compared or aggregated (Morris & DeShon, 2002, and references cited therein). Finally, meta-analysis is impossible when studies have measured different parts of a larger overarching hypothesis. Such a situation is illustrated by Kevenaar et al. (2021), who investigated children’s self-control ratings obtained from multiple informants across four different cohorts. The authors wanted to test the overarching hypothesis that children themselves report most problem behaviors, followed by their mothers and fathers, and that teachers report the fewest problems. However, in each cohort, ratings from only two or three (non-overlapping) informant groups were available, making it impossible to investigate the hypothesis of interest using meta-analysis.

In situations where meta-analysis is difficult or impossible, Bayesian evidence synthesis (BES) may provide a feasible and flexible alternative (Klugkist & Volker, 2023; Kuiper et al., 2013). As we will further elaborate below, BES consists of three steps. First, in each study, statistical hypotheses are formulated that reflect the theories of interest, but that incorporate data and design characteristics unique to that study. Then, Bayes factors are computed to quantify the evidence for the hypotheses in that study. Finally, the study-specific Bayes factors are aggregated to determine which hypothesis best accounts for each study’s results when all studies are considered simultaneously. Crucially, BES poses less constraints on the studies to be combined than meta-analysis because studies are combined at the hypothesis level rather than at the level of the effect size. The effect sizes across studies therefore do not need to have the same statistical form. In addition, BES allows for differences in study design and operationalization of variables, as the study-specific hypotheses do not have to be identical. The key idea is that if the study-specific hypotheses test the same underlying (i.e., latent) effect, the Bayes factors for these hypotheses can be meaningfully combined. BES is thus a flexible tool that allows the aggregation of highly diverse studies. However, in contrast to meta-analysis, BES is solely concerned with hypothesis testing. It does not allow researchers to estimate the size of an effect or test whether there is systematic heterogeneity in effect sizes across studies. In addition, unlike meta-analysis, BES is not intended to increase the statistical power for detecting an effect (as will become clear in the remainder of this paper and is also explained in Klugkist and Volker, 2023). For these reasons, meta-analysis will be the preferred method when studies have similar designs and measures. However, hypotheses can often be tested in many different ways (e.g., experiments, tests, surveys, vignette studies) with many different models or parameter estimates (e.g., logistic regression, multilevel models, ANOVA). Hypotheses are often assumed to hold regardless of these (sometimes arbitrary) design choices, as long as all outcomes are considered to measure the same underlying (latent) construct and the sample is considered to be drawn from the population of interest. In such situations, BES can provide us with the global support for each hypothesis across all available studies. In addition, as we will further explain below, BES allows for (i) formulating and testing informative hypotheses that unlike the conventional null hypothesis can directly test a specific hypothesis, and (ii) evaluating multiple (i.e., 2+) hypotheses simultaneously, which allows researchers to directly compare all hypotheses of interest.

The goal of the current paper is to introduce BES as an alternative to meta-analysis when the latter is difficult or impossible and to assess how BES performs in comparison to meta-analysis under various conditions. This will help researchers who are familiar with meta-analysis to correctly evaluate BES results and to understand when and why these results may diverge from what would be expected in a meta-analysis. To investigate the performance of BES in comparison to meta-analysis, we conducted a Monte Carlo simulation study in which we mimicked various scenarios that may be relevant to applied researchers. To also provide readers with a real-world example, we included an empirical demonstration in which we applied BES to a published meta-analysis on statistical learning in people with and without developmental language disorder (Lammertink et al., 2017). Before we turn to the simulation study, we will first explain BES in more detail.

### Bayesian evidence synthesis

As mentioned above, BES proceeds in three steps: (i) formulation of study-specific hypotheses, (ii) evaluation of these hypotheses in each study separately using Bayes factors, and (iii) the aggregation of these study-specific Bayes factors to yield the support for the overall theory over all studies combined. We now explain each step in turn.

### Formulation of study-specific hypotheses

In the null hypothesis significance testing (NHST) framework, a null hypothesis (${H}_{0}$: no effect) is tested against the complement hypothesis (not ${H}_{0}$). However, in the context of BES, it is also possible to evaluate informative hypotheses that represent an explicit theory or expectation by posing constraints on model parameters (e.g., Hoijtink, 2012; Klugkist et al., 2011; van de Schoot et al., 2011). For instance, in an experimental design, specific conditions are included because it is *a priori* expected that in certain conditions participants will score higher or lower than in other conditions. This expectation can be represented by order constraints on the means and could, for example, lead to the informative hypothesis ${H}_{i}: {\mu }_{1}{<\mu }_{2}<{\mu }_{3}$ (where ${\mu }_{j}$ is the mean of the *j*^th^ group or condition). Finding support for ${H}_{i}$ (or not) is then more informative than the evaluation of the usual null (all means equal) and complement (not all means equal). Order constraints are just one example of useful constraints to represent specific expectations. Also fitting in the framework of informative hypothesis evaluation are equality constraints (e.g., $\mu =0.5$, or, ${\mu }_{1}={\mu }_{2}$), range constraints (e.g., $-0.1<\mu <0.1$, or, ${\mu }_{1}>({\mu }_{2}+0.5)$) and constraints on functions of parameters (e.g., ${(\mu }_{1}+{\mu }_{2})/2>{\mu }_{3}$, or, ${\mu }_{1}>3{\mu }_{2}$). A special hypothesis is the unconstrained hypothesis which poses no constraints on the parameters (in the examples above, this means that all means can take on any value). As we explain below, this hypothesis is used in the computation of Bayes factors comparing two informative hypotheses. In addition, the unconstrained hypothesis is often included in the set of hypotheses under consideration to avoid choosing between different competing hypotheses when none of these hypotheses represent the data well (Hoijtink et al., 2019).

In the context of BES, it is furthermore possible to formulate study-specific hypotheses that test the overarching theory while also incorporating data characteristics and research methodology unique to that study. For example, say that two studies have investigated the effect of age on willingness to take risks where one study has measured the participant’s age in years and the other study has divided the participants into three age groups. The overarching hypothesis is that age decreases the willingness to take risks. This theory can be translated into the study-specific hypothesis ${H}_{i,1}: {\beta }_{age}<0$ in Study 1, where ${\beta }_{age}$ is the regression estimate for the effect of age on willingness of taking risks; and into ${H}_{i,2}: {\mu }_{1}>{\mu }_{2}>{\mu }_{3}$ in Study 2, where ${\mu }_{j}$ is the mean willingness to take risks in the *j*th age group. Another example is provided by the study of Kevenaar et al. (2021) that was briefly mentioned above, where the authors wished to aggregate four cohort studies that provided ratings of children’s self-control problems by different informants. One of the overarching hypotheses the authors wished to test was ${H}_{i}: {\mu }_{self}>{\mu }_{mother}>{\mu }_{father}>{\mu }_{teacher}$. However, as the cohort studies obtained ratings from different sets of informants, the authors specified three different study-specific hypotheses, namely ${H}_{i,1}: {\mu }_{mother}>{\mu }_{father}>{\mu }_{teacher}$; ${H}_{i,2}: {\mu }_{self}>{\mu }_{mother}$; and ${H}_{i,3}: {\mu }_{mother}>{\mu }_{teacher}$. The idea here is that these study-specific hypotheses should receive the most support in each study if ${H}_{i}$ is true because these hypotheses are all compatible with ${H}_{i}$ even though they only test part of it.

### Hypothesis evaluation using the Bayes factor

The relative support for a given hypothesis (or model) can be expressed with the Bayes factor (BF). Note that in the Bayesian testing framework, a hypothesis is formulated as a statistical model; throughout the remainder of this paper we will therefore use the terms *hypothesis* and *model* interchangeably. The BF comparing hypotheses ${H}_{i}$ and ${H}_{i^{\prime}}$ is given by

1 $${BF}_{ii^{\prime}}=\frac{P({\varvec{X}}|{H}_{i})}{P({\varvec{X}}|{H}_{i^{\prime}})}=\frac{\int P\left({\varvec{X}}|{\varvec{\beta}},{H}_{i}\right)P\left({\varvec{\beta}}|{H}_{i}\right) \partial{\varvec{\beta}}}{\int P\left({\varvec{X}}|{\varvec{\beta}},{H}_{i^{\prime}}\right)P\left({\varvec{\beta}}|{H}_{i^{\prime}}\right) \partial{\varvec{\beta}}} ,$$

where $P({\varvec{X}}|{H}_{i})$ and $P({\varvec{X}}|{H}_{i^{\prime}})$ denote the marginal likelihood of the observed data under hypothesis ${H}_{i}$ and ${H}_{i^{\prime}}$, respectively (Kass & Raftery, 1995). These marginal likelihoods are defined as the product of the likelihood function, $P\left({\varvec{X}}|{\varvec{\beta}}, H\right)$, and the prior, $P\left({\varvec{\beta}}|H\right)$, integrated with respect to the parameter vector ${\varvec{\beta}}$. The BF can be directly interpreted as the evidence in the data for hypothesis ${H}_{i}$ versus the evidence in the data for hypothesis ${H}_{i^{\prime}}$. As such, a ${BF}_{ii^{\prime}}=10$ for example indicates that hypothesis ${H}_{i}$ receives 10 times more support than hypothesis ${H}_{i^{\prime}}$.

Calculation of the BF based on its mathematical definition presented in Eq. 1 is typically difficult. However, building on work by Klugkist et al. (2005), Gu et al. (2018) showed that the BF comparing a hypothesis ${H}_{i}$ to the unconstrained hypothesis ${H}_{u}$ can be approximated by the Savage-Dickey density ratio in Eq. 2 for equality-constrained hypotheses (e.g., $\beta =0$) when (i) using normal approximations of the prior and posterior distributions of the unconstrained hypothesis, (ii) centering the prior distribution on the boundary of the hypotheses under consideration, and (iii) using a fraction *b* of the information in the data to construct a proper prior distribution. This yields

2 $${BF}_{{i}_{0}u}=\frac{{f}_{{i}_{0}}}{{c}_{{i}_{0}}} = \frac{{P}_{u}({\varvec{\beta}}={{\varvec{B}}}_{{{\varvec{i}}}_{0}}|{\varvec{X}})}{{p}_{u}^{*}({\varvec{\beta}}={{\varvec{B}}}_{{{\varvec{i}}}_{0}}|{{\varvec{X}}}^{b})} ,$$

where ${f}_{{i}_{0}}$ is the density of the unconstrained posterior distribution ${P}_{u}({\varvec{\beta}}={{\varvec{B}}}_{{{\varvec{i}}}_{0}}|{\varvec{X}})$ (denoted fit) and ${c}_{{i}_{0}}$ is the density of the adjusted unconstrained prior distribution ${p}_{u}^{*}({\varvec{\beta}}={{\varvec{B}}}_{{{\varvec{i}}}_{0}}|{{\varvec{X}}}^{b})$ (denoted complexity) evaluated at the location of the hypothesized values ${{\varvec{B}}}_{{{\varvec{i}}}_{0}}$.

For inequality-constrained hypotheses (e.g., $\beta >0$), ${BF}_{iu}$ can be approximated by

3 $${BF}_{{i}_{1}u}=\frac{{f}_{{i}_{1}}}{{c}_{{i}_{1}}} = \frac{{\int }_{{\varvec{\beta}}\in {{\varvec{B}}}_{{{\varvec{i}}}_{1}}}{P}_{u}({\varvec{\beta}}|{\varvec{X}})\partial{\varvec{\beta}}}{{\int }_{{\varvec{\beta}}\in {{\varvec{B}}}_{{{\varvec{i}}}_{1}}}{p}_{u}^{*}\left({\varvec{\beta}}|{{\varvec{X}}}^{b}\right) \partial{\varvec{\beta}}} ,$$

where the fit (${f}_{{i}_{1}}$) is the proportion of the unconstrained posterior distribution that is in line with hypothesis ${H}_{i}$, and the complexity (${c}_{{i}_{1}}$) is the proportion of the unconstrained prior distribution for ${H}_{u}$ in line with hypothesis ${H}_{i}$. For the computation of ${BF}_{iu}$ for hypotheses with both equality and inequality constraints see Gu et al. (2018, p. 241).

The BF comparing two informative hypotheses ${H}_{i}$ and ${H}_{i^{\prime}}$ is then given by

4 $${BF}_{ii^{\prime}}=\frac{{BF}_{iu}}{{BF}_{i^{\prime}u}}=\frac{{f}_{i}/{c}_{i}}{{f}_{i^{\prime}}/{c}_{i^{\prime}}} .$$

The support expressed by the BF thus balances the fit and complexity of the hypotheses under consideration. The fit of a hypothesis is a measure of how well the hypothesis describes the observed data, while the complexity indicates how specific (parsimonious) the hypothesis is. The higher the fit, and the lower the complexity, the higher the BF in favor of the hypothesis at hand relative to an alternative hypothesis. This means that whenever two hypotheses have an equal fit, the most parsimonious hypothesis will be preferred. However, even when a given hypothesis ${H}_{i}$ has a *lower* fit than an alternative hypothesis ${H}_{i^{\prime}}$, ${H}_{i}$ will still be preferred if the decrease in complexity for ${H}_{i}$ compared to ${H}_{i^{\prime}}$ is larger than the decrease in fit. When evaluating the set of hypotheses under consideration, it is thus important to consider how the relative complexities of these hypotheses will influence the results. The most specific (least complex) hypotheses are equality-constrained hypotheses (e.g., ${\mu }_{1}={\mu }_{2}$). The least specific (most complex) hypothesis is the unconstrained hypothesis ${H}_{u}$ which poses no constraints on the parameter values. The unconstrained hypothesis will therefore only be preferred if none of the other hypotheses provide a good fit to the data. Note that per their definition, the complexity and fit of ${H}_{u}$ are always 1, which means there is an upper limit of ${BF}_{iu}$ that is determined by the complexity of ${H}_{i}$ (Klugkist & Volker, 2023). For example, the hypothesis ${H}_{1}: \beta >0$ has a complexity of 0.5 since this hypothesis covers half of the unconstrained prior distribution. This means that ${BF}_{1u}$ has an upper limit of 2: when the fit of ${H}_{1}$ is perfect, ${BF}_{1u}=1/0.5=2$. In contrast, when testing a hypothesis against its complement or against another constrained hypothesis, the resulting BF does not have an upper limit. If a researcher is interested in only one informative hypothesis, then testing against the complement is the most powerful, because the two hypotheses cover mutually exclusive regions of the parameter space (Klugkist & Volker, 2023). Note, finally, that when testing an equality-constrained hypothesis (e.g., ${H}_{0}: {\mu }_{1}={\mu }_{2}$), the unconstrained hypothesis ${H}_{u}$ is statistically equivalent to the complement hypothesis ${H}_{c}$ (not ${H}_{0}$; Hoijtink et al., 2019).^1^ We will further illustrate the interplay between the fit and complexity of the hypotheses under consideration in the simulation results.

When comparing a set of hypotheses, it is useful to translate the BFs into posterior model probabilities (PMPs; Kass & Raftery, 1995). PMPs facilitate interpretation as they have values between 0 and 1 (with values closer to 1 indicating more support) that add up to 1 over all hypotheses under consideration; they thus express the relative support for each of the tested hypotheses. Translating BFs into PMPs is simple, given that the BF is a multiplicative factor that transforms the prior odds of two hypotheses (i.e., the ratio of the probabilities of each hypothesis before any data is collected) into the posterior odds (i.e., the ratio of the probabilities of each hypothesis after seeing the data), as shown in Eq. 5:

5 $$\frac{P({H}_{i})}{P({H}_{u})}\times {BF}_{iu}= \frac{P({H}_{i}|{\varvec{X}})}{P({H}_{u}|{\varvec{X}})}.$$

The PMP for hypothesis ${H}_{i}$ can thus be computed as

6 $$PMP({H}_{i})=\frac{P({H}_{i})\times {BF}_{iu}}{\sum_{i=1}^{m}P({H}_{i})\times {BF}_{iu}} ,$$

where $P({H}_{i})$ is the prior model probability of hypothesis ${H}_{i}$ with *i* = 1, 2, …, *m*. Typically, equal prior probabilities are assigned to each hypothesis, which means that each hypothesis receives a prior model probability of 1/*m*.

### Synthesis of Bayes factors

Once the study-specific BFs (or PMPs) are obtained, the final step is to aggregate them to yield the joint support for each hypothesis across all studies. The joint support for each hypothesis is obtained by updating the model probabilities with each new study. In other words, the posterior model probability of study *k* can be used as the prior model probability for study *k*+1. Irrespective of the order of the studies, this process can be repeated for a total of *K* studies, assuming all studies are independent (Kuiper et al., 2013). The aggregated PMP for hypothesis ${H}_{i}$ is then given by

7 $${PMP({H}_{i})}^{K}=\frac{{P}^{0}({H}_{i})\times \prod_{k=1}^{K}{BF}_{iu}^{k}}{\sum_{i=1}^{m}{P}^{0}\left({H}_{i}\right)\times \prod_{k=1}^{K}{BF}_{iu}^{k}} ,$$

where ${P}^{0}({H}_{i})$ indicates the prior model probability for hypothesis ${H}_{i}$ before any study has been conducted. The numerator represents the joint probability of the data from all studies under the assumption that the constraints of the target hypothesis hold separately in each study, whereas the denominator sums the joint probabilities of the data under each of the hypotheses under consideration. The aggregated PMP therefore provides the joint evidence for a hypothesis in each study relative to the other hypotheses considered. Note that in order to compute the aggregated PMP it is not necessary for the study-specific BFs to have used the same priors for the *model* *estimates*. BES assumes that studies provide independent pieces of evidence, which means that if the prior used within a study is deemed appropriate to estimate the parameters and/or compute the BFs, then the evidence from this study can be aggregated with the evidence from other studies regardless of whether these other studies used the same prior on the model estimates (see Klugkist & Volker, 2023).

With equal prior *model probabilities* for each hypothesis (i.e., ${P}^{0}({H}_{i})$ = 1/*m*), Eq. 7 can be rewritten as

8 $${PMP({H}_{i})}^{K}=\frac{\prod_{k=1}^{K}{PMP({H}_{i})}^{k}}{\sum_{i=1}^{m}\prod_{k=1}^{K}{PMP({H}_{i})}^{k}} ,$$

where ${PMP({H}_{i})}^{k}$ is the posterior model probability of hypothesis ${H}_{i}$ in study $k$. When only two hypotheses are tested, this formula simplifies to

9 $${PMP({H}_{i})}^{K}=\frac{\prod_{k=1}^{K}{PMP({H}_{i})}^{k}}{\prod_{k=1}^{K}{PMP({H}_{i})}^{k}+ {\prod }_{k=1}^{K}{(1-PMP({H}_{i})}^{k})} .$$

Note that with equal prior model probabilities, the information provided by the aggregated PMP is the same as the product of Bayes factors.

### Difference with meta-analysis

As mentioned above, the aggregated PMPs obtained by BES give the relative joint probability of the data from all studies under the assumption that the constraints of the target hypothesis hold in each study separately. The aggregated PMP can therefore be used to indicate which hypothesis best describes each study. This is different from inferences based on data-pooling techniques such as meta-analysis, which indicate whether the target hypothesis is supported by the *pooled* data. In a meta-analysis (e.g., Hedges & Olkin, 1985; Hedges & Vevea, 1998), it is assumed that for a set of $k=1,\dots ,K$ independent studies, the observed effect in study $k$ is given by

10 $${y}_{k}\sim N\left({\theta }_{k}, {v}_{k}\right),$$

where ${\theta }_{k}$ is the (unknown) true effect, and ${v}_{k}$ is the sampling variance (which is assumed to be known). Since most meta-analyses are based on sets of studies that are not identical, it is typically assumed that there is variability among the true effects. If this variability is not systematic (i.e., between-study differences do not systematically predict effect size), then this variability can be modeled as purely random with the random-effects model given by

11 $${\theta }_{k} \sim N\left(\mu , {\tau }^{2}\right),$$

that is, the true effects ${\theta }_{k}$ are assumed to be normally distributed with mean $\mu$ and variance ${\tau }^{2}$. In contrast to BES, where the model parameters are estimated independently in each study and are therefore allowed to vary, meta-analysis assumes that the mean population effect $\mu$ is identical for each study. To yield a better estimate of this common population parameter, a weighted average of the observed effect sizes ${y}_{k}$ is computed, with weights typically equal to the inverse variance (i.e., $1/[{v}_{k}+ {\widehat{\tau }}^{2}]$, where ${\widehat{\tau }}^{2}$ denotes the estimate of ${\tau }^{2}$). Inferential tests and confidence intervals then indicate whether the estimated common population parameter significantly differs from zero.

It can sometimes happen that BES does not yield the same results as data-pooling techniques like meta-analysis, as for example illustrated by Regenwetter et al. (2018), who used both BES (which they called the “group BF”) and a data-pooling technique (i.e., BFs computed on the pooled data which they called the “pooled BF”) in the context of aggregating single participant data. Diverging results may, for example, occur when the hypothesis best supported by the aggregated data is not well supported in any of the individual studies. Another example of when the methods may not converge on the same hypothesis is when a given hypothesis, ${H}_{i}$, describes most studies reasonably well but provides a *very poor* fit for a few studies that are better described by other hypotheses. In this case, ${H}_{i}$ will typically not be selected as the best hypothesis by BES but might still be selected as the best hypothesis by data-pooling techniques. Although both meta-analysis and BES can thus be used for testing hypotheses across multiple studies, the results may sometimes differ because the methods answer a different synthesis question. This will be further illustrated in the simulation study.

## Simulation

In the simulation study, we evaluated the performance of BES compared to meta-analysis as a function of true population effect size, total sample size per study, level of variability among the study-specific true effects, and number of studies. We also assessed how much influence one study with an extremely small sample size or opposite effect size has on the aggregated result. For BES, we show how the results depend on which hypotheses are considered. The simulation was conducted in R (R Core Team, 2021, Version 4.1.0). All R scripts, simulated datasets, and (supplementary) figures are available in the Open Science Framework repository at https://osf.io/gbtyk/.

### Data generation

We used the standardized mean difference as an effect size as this is a very common effect-size metric in meta-analyses of experimental studies. For each artificial study, $k$, with $k=1,\dots ,K$, we generated data for a total of $N$ participants divided equally across two groups, which we refer to here as the experimental group and the control group. Let ${\mathbf{Y}}_{k}^{E}$ be the $N/2\times 1$ vector of outcomes for the experimental group and ${\mathbf{Y}}_{k}^{C}$ the $N/2\times 1$ vector of outcomes for the control group. Assuming normality of the data, these outcomes can be generated as

12 $${\mathbf{Y}}_{k}^{E} \sim N\left({\delta }_{k}, 1\right)\mathrm{ and }\;{\mathbf{Y}}_{k}^{C} \sim N\left(0, 1\right),$$

where ${\delta }_{k}$ is the true standardized mean difference for study $k$. Given the relatively large amount of heterogeneity in effect sizes found in psychological meta-analyses (Linden & Hönekopp, 2021; van Erp et al., 2017), we modeled variability among the study-specific true effect sizes by sampling them from a normal distribution, that is,

13 $${\delta }_{k} \sim N\left(\delta ,{\tau }^{2}\right),$$

where $\delta$ is the true mean population effect size and ${\tau }^{2}$ is the true between-study variance in effect sizes. After simulating the data, we estimated the standardized mean difference ${\widehat{\delta }}_{k}$ and its variance ${v}_{k}$ following standard formulas, that is,

14 $${\widehat{\delta }}_{k}=\frac{{\overline{Y} }_{k}^{E}- {\overline{Y} }_{k}^{C}}{\sqrt{{(SD}_{1}^{2}+{SD}_{2}^{2})/2} }$$

and

15 $${v}_{k}= \frac{4}{N}+ \frac{{\widehat{\delta }}_{k}^{2}}{2(N-2)} ,$$

The different simulation conditions were created by varying the true mean population effect size ($\delta$), the true between-study standard deviation ($\tau$), and the total sample size per study ($N$). For $\delta ,$ we chose a small ($\delta =0.2$) and medium effect ($\delta =0.5$), given that small-to-medium effect sizes are the most common in the field of psychology (Lovakov & Agadullina, 2021; Open Science Collaboration, 2015). We also included a null effect ($\delta =0)$ to investigate the behavior of BES when the null hypothesis is true. For $\tau$, we chose the first ($\tau =0.1)$ and third quartile ($\tau =0.3$) of estimated $\tau$ values found in 189 meta-analyses of standardized mean differences published in *Psychological Bulletin* (van Erp et al., 2017), thus representing relatively small and relatively large variation in effect sizes within the field of psychology. Finally, for $N$ we chose a range between 20 and 200 with a step size of 20. The lower limit represents the absolute minimal sample size typically considered for experimental studies in psychology, whereas the upper limit represents the maximum sample size found in ~ 80% of 2642 experimental studies collected by Lovakov & Agadullina (2021). For each of the 3 ($\delta \in \{0, 0.2, 0.5\}$) x 2 ($\tau \in \{0.1, 0.3\}$) x 10 ($N\in \{20, 40, \dots , 200\}$) = 60 conditions, we simulated a set of $K=30$ studies. We ran $1000$ replications per condition, yielding a total of 60 x 30 x 1000 = 1,800,000 simulated datasets. To investigate the effect of the number of studies, we synthesized the first two studies of each replication by means of meta-analysis and BES and then cumulatively added one study at a time until all $K=30$ studies were synthesized. This way, we could directly investigate the effect of adding more studies to the existing set of studies.^2^

We also investigated how many studies one needs to still yield aggregated support for the target hypothesis  when one study provides strong evidence *against* this hypothesis. This may for example happen when one of the studies is underpowered (and therefore provides more support for the null hypothesis), or when one of the studies is sampled from a population with an opposite effect size (e.g., when this is the only study investigating older participants, and the effect turns out to be reversed for older vs. younger participants). To investigate this, we focused on the subset of studies with $\delta =0.5$ and $N=140$, such that all studies had sufficient power to detect the true population effect; detecting a standardized mean difference of 0.5 with 80% power requires a total sample size of 128 (calculation performed with G*power; Faul et al., 2007). Then, we replaced the first study in each replication by a newly simulated study with a total sample size of $N=30$ (representing an underpowered study) or by a newly simulated study with a population effect of $\delta =-0.5$ (representing a study that tested a sample from a different population).

### Meta-analysis

Given that effect sizes in psychology are typically assumed to be heterogenous, we conducted a random-effects meta-analysis (henceforth meta-analysis) with the widely used rma() function from the *metafor* package (Viechtbauer, 2010). This function fits a random-effects model using a two-step approach. First, the amount of between-study variance (i.e., ${\tau }^{2}$) is estimated using a restricted maximum likelihood estimator (Raudenbush, 2009; Viechtbauer, 2005). Then, the average true effect is estimated via weighted least squares, with weights equal to the inverse variance. Once the parameter estimates have been obtained, confidence intervals are computed based on a standard normal distribution.

### Bayesian evidence synthesis

For BES, we formulated our hypothesis of interest as ${H}_{1}: \delta >0$. This hypothesis can be tested against three possible alternative hypotheses, namely the null hypothesis, ${H}_{0}:\delta =0$, the complement hypothesis, ${H}_{c}$: not ${H}_{1}$ (in this case $\delta <0$), and the unconstrained hypothesis, ${H}_{u}:\delta$ (i.e., $\delta$ can take on any value). We tested ${H}_{1}$ against each of these alternative hypotheses in turn to demonstrate how the individual characteristics of each alternative hypothesis affect the results. In practice, however, researchers may often want to test multiple hypotheses simultaneously (for examples of empirical studies that used BES to test multiple hypotheses simultaneously, see Kevenaar et al., 2021; Veldkamp et al., 2021; Zondervan‐Zwijnenburg, Richards et al., 2020a, Zondervan-Zwijnenburg, Veldkamp et al., 2020b) and we will show how to do this in the empirical demonstration. Nevertheless, it may sometimes happen that researchers are *only* interested in testing a hypothesis ${H}_{i}$ against ${H}_{c}$, for example when there are two competing theories that make opposite predictions, and the null hypothesis is considered very unlikely. Likewise, a researcher may opt to only test ${H}_{i}$ against ${H}_{u}$ when ${H}_{i}$ is the only theoretically relevant hypothesis.

To obtain the study-specific PMPs for ${H}_{1}$ we used the R-package *bain* (Gu et al., 2020), which computes the approximate adjusted fractional BFs given in Eqs. (2–4) based on the effect size estimates (${\widehat{\delta }}_{k}$), their variance (${v}_{k}$) and the study-specific sample size, and translates these BFs into PMPs (see Eq. 6). We assumed equal prior probabilities for each hypothesis and used bain’s default priors for the effect-size estimates. After computing the study-specific PMPs, we calculated the aggregated PMPs according to Eq. (9).

### Performance measure

We compared meta-analysis and BES by looking at the proportion of times ${H}_{1}$ was “accepted” across simulations. In theory, we cannot accept ${H}_{1}$ within the frequentist framework (we can only reject ${H}_{0}$), but we considered the meta-analysis results to be compatible with ${H}_{1}$ if the meta-analytic effect size was positive and the lower bound of the confidence interval (CI) was greater than zero. We calculated the ${H}_{1}$ acceptance rate based on both 95% CIs (as this is what is typically reported in the literature) as well as 90% CIs (given that ${H}_{1}$ is a directional hypothesis).

For BES, we adopted the decision rule used by Klaassen et al. (2018) who considered a hypothesis ${H}_{i}$ the best of a set of $m$ hypotheses if the evidence for ${H}_{i}$ was at least $m-1$ times (with a minimum value of 2) stronger than for any other hypothesis. This ensures that the aggregated PMP of the best hypothesis is at least .50 when all hypotheses are equally likely *a priori*. Since we only tested two hypotheses at a time, this means that we accepted ${H}_{1}$ if its aggregated PMP was at least twice as high as for the alternative hypothesis (i.e., ${BF}_{1.}=2$), corresponding to an aggregated PMP of .67 or higher for ${H}_{1}$, and .33 or lower for the alternative hypothesis.

The advantage of this decision rule is that it considers the number of hypotheses under consideration. The larger the number of hypotheses, the less support any one hypothesis will receive (Hoijtink et al., 2019), meaning that using the same cut-off point for comparing two or three (or more) hypotheses is not appropriate. However, which value of the aggregated PMP can be seen as “strong” evidence remains a question for future research and will likely vary as a function of the research field, the number of hypotheses evaluated, and the characteristics of the hypotheses under consideration. Although often-cited guidelines in the literature consider a BF of 10 to provide strong evidence (corresponding to a PMP of .91; Jeffreys, 1961), these guidelines were made for evaluating the unconstrained hypothesis against the null hypothesis in a single study and do not necessarily generalize to the context of evaluating other, or multiple, hypotheses *across* studies.

To investigate the sensitivity of the results to the employed decision rule, we also used a cut-off value of .91 for the aggregated PMP, as shown in Figs. S1 to S4 (available at https://osf.io/gbtyk/). These figures show that this higher cut-off value mainly affects the results when testing against ${H}_{u}$. We will come back to this finding in the Discussion.

## Results

**Fig. 1:**
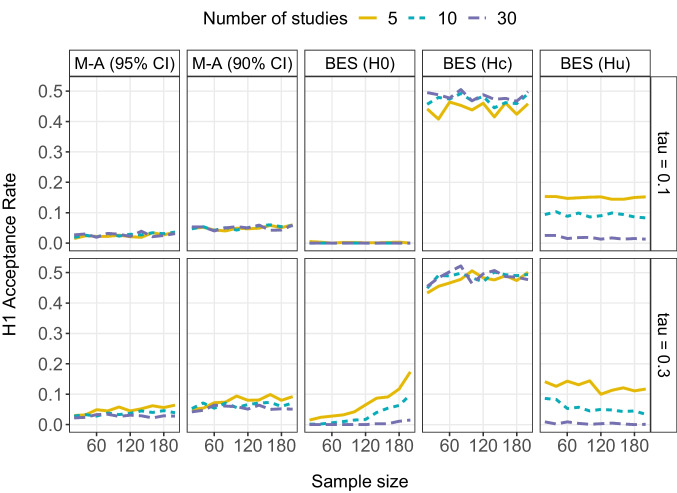
${H}_{1}$ acceptance rate for random-effects meta-analysis (M-A) with either 95% or 90% confidence intervals and Bayesian evidence synthesis (BES) as a function of the between-study standard deviation in population effect sizes $\tau$, total sample size per study, and number of studies when the overall mean population effect size is zero. Note that for BES, ${H}_{1}$ ($\delta$ > 0) is tested against three alternative hypotheses: ${H}_{0}$ ($\delta$ = 0), ${H}_{c}$ ($\delta$ < 0), or ${H}_{u}$ ($\delta$)

**Fig. 2:**
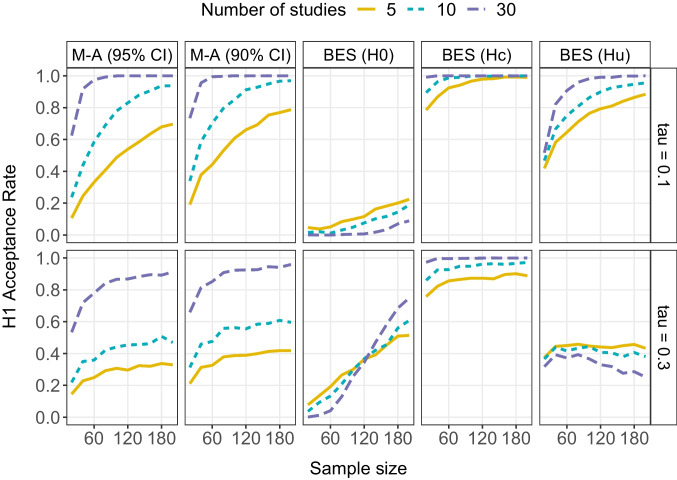
${H}_{1}$ acceptance rate for random-effects meta-(M-A) with either 95% or 90% confidence intervals and Bayesian evidence synthesis (BES) as a function of the between-study standard deviation in population effect sizes $\tau$, total sample size per study, and number of studies when the overall mean population effect size is 0.20 (i.e., a small effect). Note that for BES, ${H}_{1}$ ($\delta$ > 0) is tested against three alternative hypotheses: ${H}_{0}$ ($\delta$ = 0), ${H}_{c}$ ($\delta$ < 0), or ${H}_{u}$ ($\delta$)

**Fig. 3:**
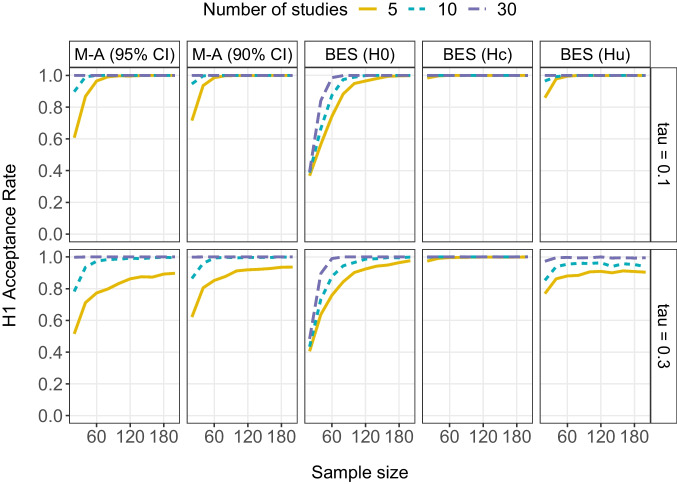
${H}_{1}$ acceptance rate for random-effects meta-analysis (M-A) with either 95% or 90% confidence intervals and Bayesian evidence synthesis (BES) as a function of the between-study standard deviation in population effect sizes $\tau$, total sample size per study and number of studies when the overall mean population effect size is 0.50 (i.e., a medium effect). Note that for BES, ${H}_{1}$ ($\delta$ > 0) is tested against three alternative hypotheses: ${H}_{0}$ ($\delta$ = 0), ${H}_{c}$ ($\delta$ < 0), or ${H}_{u}$ ($\delta$)

**Fig. 4:**
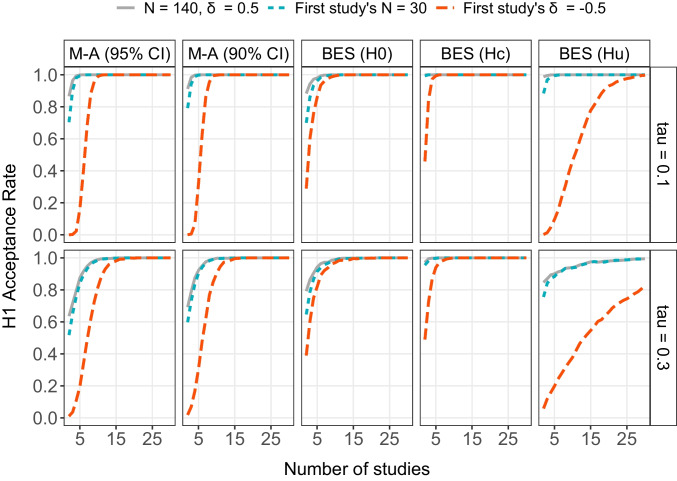
${H}_{1}$ acceptance rate for random-effects meta-analysis (M-A) with either 95% or 90% confidence intervals and Bayesian evidence synthesis (BES) as a function of the between-study standard deviation in population effect sizes $\tau$ and number of studies (between 2 and 30). The *lines* represent three scenarios: (i) all studies had an *N* of 140 and a $\delta$ of 0.5 (*grey solid line*); (ii) same as the grey line but the first study had an *N* of 30 (*blue short-dashed line*); (iii) same as the grey line but the first study had a $\delta$ of – 0.5 (*red long-dashed line*). Note that for BES, ${H}_{1}$ ($\delta$ > 0) is tested against three alternative hypotheses: ${H}_{0}$ ($\delta$ = 0), ${H}_{c}$ ($\delta$ < 0), or ${H}_{u}$ ($\delta$)

In Figs. 1, 2 and 3 we show the ${H}_{1}$ acceptance rate across simulation replications as a function of (i) the synthesis method (meta-analysis vs. BES), (ii) the total sample size per study, (iii) the between-study standard deviation $\tau$, and (iv) the number of studies synthesized. We show separate lines for the synthesis of 5, 10, and 30 studies, as these represent the first, second, and third quartile of the number of effect sizes synthesized per meta-analysis in 747 meta-analyses reported in *Psychological Bulletin* (van Erp et al., 2017).^3^ In these figures, the true mean population effect size $\delta$ is 0, 0.2 and 0.5, respectively, representing a null, small, and medium effect. Note that when the true effect is zero (Fig. 1), a low ${H}_{1}$ acceptance rate indicates better performance of the method. Finally, in Fig. 4, we focus on the subset of studies with $\delta =0.5$ and $N=140$ and show how including one underpowered study or one study with an opposite population effect influences the results.

### Null effect (${\varvec{\delta}}=0$)

Figure 1 shows the results when the null hypothesis is true. Note that in this figure the ${H}_{1}$ acceptance rate runs from 0 to 0.5, rather than to 1. As expected, ${H}_{1}$ is accepted in less than 5% of the meta-analyses based on 95% CIs, and less than 10% of the meta-analyses based on 90% CIs, irrespective of sample size, number of studies and $\tau$ values (although the ${H}_{1}$ acceptance rate slightly increases when between-study variance is large and few studies are combined). For BES, the results depend on the alternative hypothesis. When testing against ${H}_{0}$, ${H}_{1}$ is consistently rejected when there is relatively little variation (i.e., $\tau$ = 0.1) in population effect sizes across studies. In contrast, when there is relatively large variation in population effect sizes (i.e., $\tau$ = 0.3), the ${H}_{1}$ acceptance rate slightly increases with larger sample sizes, especially when combining less than 30 studies. This can be explained as follows: When the between-study variance in true effect sizes is large, there will be more studies with true-positive effects as well as with true-negative effects. Positive effects support the hypothesis of interest ${H}_{1}$ (especially when the sample size is large), whereas (large) negative effects do not support the alternative hypothesis ${H}_{0}.$ Therefore, the ${H}_{1}$ acceptance rate increases with larger sample sizes when there is large between-study variation among the true effects. However, this trend is countered by combining more studies, as the true effect of most studies will be near the mean population effect size ($\delta =0$). With more studies, the aggregated support for ${H}_{0}$ will eventually outweigh the few studies that support ${H}_{1}$.

When testing ${H}_{1}$ against ${H}_{u}$, we expect ${H}_{u}$ to receive more support than ${H}_{1}$ when ${H}_{1}$ is not true, as is the case here. We see that ${H}_{1}$ is indeed rejected at least 80% of the time when we combine five studies, and almost 100% of the time when we combine 30 studies. When we use a higher cut-off value for accepting ${H}_{1}$, we reject ${H}_{1}$ more often: When the cut-off value is .91, we consistently reject ${H}_{1}$ regardless of $\tau$ value, sample size or number of studies combined (see Fig. S1 at https://osf.io/gbtyk/).

Finally, when testing ${H}_{1}$ against ${H}_{c}$, the ${H}_{1}$ acceptance rate is around .50, which is to be expected since the true mean population effect (i.e., $\delta =0$) is on the boundary of the considered hypotheses. This shows the importance of including all relevant hypotheses: When the true hypothesis (in this case *H*_0_) is not in the set of considered hypotheses, one runs the risk of selecting a hypothesis that provides a poor fit to the data.

### Small effect (${\varvec{\delta}}=0.2$)

Figure 2 shows the results when the mean population effect is small (i.e., $\delta =0.2$). Note that this represents a situation in which all studies are underpowered, as detecting a standardized mean difference of 0.2 with 80% power would require a total sample size of 788 per study (calculation performed with G*power; Faul et al., 2007). Given the prevalence of small-to-medium effects and the rarity of sample sizes over 200, this is expected to be a typical situation in the field of experimental psychology (see Linden & Hönekopp, 2021; Lovakov & Agadullina, 2021; Open Science Collaboration, 2015).

When the mean population effect is small, the ${H}_{1}$ acceptance rate is rather low for meta-analysis when combining less than 30 studies, especially when variation among the true effects is large. However, for both small and large $\tau$ values, the ${H}_{1}$ acceptance rate increases when combining more studies. This exemplifies how meta-analysis helps mitigate power issues, especially when combining a relatively large number of studies.

For BES, the results again depend on the alternative hypothesis. When testing against ${H}_{0}$ and between-study variation is small, the ${H}_{1}$ acceptance rate is very low. This is because underpowered studies each provide stronger evidence for ${H}_{0}$ than for ${H}_{1}$, and BES answers the question of which hypothesis best describes each individual study, rather than which hypothesis best describes the pooled data. Moreover, combining more studies only strengthens, rather than weakens, the support for ${H}_{0}$, as the more studies we combine that all show support for ${H}_{0},$ the more confident we become that ${H}_{0}$ best describes each individual study (Klugkist & Volker, 2023). In other words, BES does not solve power issues when testing against the null. That said, the ${H}_{1}$ acceptance rate does slightly increase with larger sample sizes, even though studies with $N=200$ are still very underpowered (recall that a minimum sample size of $N=788$ is needed here).

A different picture emerges for testing against ${H}_{0}$ when between-study variation is large. Here we see that the ${H}_{1}$ acceptance rate increases more steeply as a function of sample size. As in Fig. 1, this is because large between-study variance in true effects yields effect sizes that are further from the mean effect in both directions, and positive effects provide support for ${H}_{1}$ whereas (large) negative effects do not provide evidence for ${H}_{0}$. We also see that when $\tau$ is large, support for ${H}_{1}$ starts to increase again with a greater number of studies once the study-specific sample sizes become large enough (i.e., when $N>120$).

When testing against ${H}_{c}$, BES consistently yields evidence in favor of ${H}_{1}$, almost regardless of sample size or the number of studies combined. This is not surprising, given that positive effects will always render more evidence for ${H}_{1}$ (i.e., $\delta$ > 0) than for ${H}_{c}$ (i.e., $\delta$ < 0), even when they are small. This shows that testing against ${H}_{c}$ is a very powerful strategy whenever the null hypothesis is not of interest or is considered very unlikely.

Finally, testing against ${H}_{u}$ yields a similar pattern of results as meta-analysis when between-study variation is small, in that the ${H}_{1}$ acceptance rate quickly increases as a function of sample size and the number of studies (with the exact ${H}_{1}$ acceptance rate depending on the employed decision rule, cf. Fig. 2 and Fig. S2). In contrast, the ${H}_{1}$ acceptance rate remains low when between-study variation is large, as large variation around a small mean effect will yield at least some studies that provide very strong evidence *against* ${H}_{1}$. In contrast, *all* studies are in line with ${H}_{u}$ by definition. ${H}_{u}$ will therefore typically receive more evidence than ${H}_{1}$ when the true mean effect is small and between-study variance is large. Moreover, the aggregated support for ${H}_{1}$ decreases, rather than increases, with the number of studies in this situation; with more studies, there is a higher chance that some of these studies provide strong evidence against ${H}_{1}$, which decreases the aggregated support for ${H}_{1}$.

### Medium effect (${\varvec{\delta}}=0.5$)

Figure 3 shows the results when all studies are drawn from a population with a medium mean effect (i.e., $\delta =0.5$). Here, we see that the ${H}_{1}$ acceptance rate is very high across all synthesis methods. We still need some power in the individual studies when testing against ${H}_{0}$ with either meta-analysis or BES, but once the total sample size per study is larger than 60, the ${H}_{1}$ acceptance rate is above .80 regardless of between-study variation, alternative hypothesis tested, or number of studies combined.

### Effect of one study with a small sample size or opposite effect size

In Fig. 4, we show the subset of studies for which $\delta =0.5$ and $N=140$ (grey line) and compare this to the same subset of studies for which the first study is replaced by a newly simulated study with a very small total sample size (i.e., $N=30$, blue line) or with an opposite mean population effect (i.e., $\delta =- 0.5$, red line). Whereas including one underpowered study hardly has an effect on the ${H}_{1}$ acceptance rate (and no effect whatsoever when combining at least five studies), including a study with an opposite population effect drastically decreases the ${H}_{1}$ acceptance rate for both meta-analysis and when testing against ${H}_{u}$ with BES (but not when testing against ${H}_{0}$ or ${H}_{c}$). This effect is countered, however, by combining more studies.

For meta-analysis, approximately ten studies that support ${H}_{1}$ need to be included to again yield aggregated support for ${H}_{1}$ at least 80% of the time. When testing against ${H}_{u}$ with BES, one needs to include at least 15 studies that support ${H}_{1}$ when between-study variation is small, and at least 30 studies when between-study variation is large (more studies are needed when using a higher cut-off value for accepting ${H}_{1}$, see Fig. S4). In other words, testing against ${H}_{u}$ is the most sensitive to including studies that show strong support *against* the hypothesis of interest. Again, this is not surprising given that ${H}_{u}$ is always true and therefore describes each study well, whereas ${H}_{1}$ in this case provides a very poor fit to one of the included studies. However, since ${H}_{1}$ is more parsimonious than ${H}_{u}$, we eventually still obtain more aggregated support for ${H}_{1}$ if we combine enough studies.

## Empirical demonstration

The data for this demonstration come from a meta-analysis on auditory verbal statistical learning in people with and without developmental language disorder (DLD), previously known as specific language impairment (SLI; Lammertink et al., 2017). In this study, ten effect sizes from eight studies were analyzed by means of a random-effects meta-analysis to investigate whether people with DLD show a statistical learning deficit compared to people without DLD. An important feature of this meta-analysis was that only effect sizes were included that came from non-overlapping participant samples. This was important given that BES relies on the assumption that studies provide independent pieces of evidence for the hypotheses under consideration.

Prior to conducting the meta-analysis, Lammertink et al. (2017) computed a standardized mean difference (SMD) for each study based on summary or test statistics reported in the primary studies (i.e., means and standard deviations per group or an *F*-/*t*-statistic). The overall standardized mean difference between participants with and without DLD was 0.54, which was significantly different from zero (*p* < .001, 95% confidence interval [0.36, 0.71]). The authors therefore concluded that there is a robust difference between people with and without DLD in their detection of statistical regularities in the auditory input, congruent with the hypothesis that people with DLD are less effective in statistical learning.

Below we demonstrate how BES can be performed on the effect sizes included in this meta-analysis. For illustrative purposes, we show how to test ${H}_{1}$ (SMD > 0; that is, participants without DLD score higher on statistical learning than participants with DLD) against each alternative hypothesis (${H}_{0}$, ${H}_{c}$, ${H}_{u}$) in turn, like we did in the simulations. Afterwards we show how multiple hypotheses can be tested simultaneously and how a different type of hypothesis (i.e., a range-constrained hypothesis) can be formulated. Note, however, that if we were to perform BES on this set of studies for an empirical paper, we would probably only test ${H}_{1}$ against ${H}_{0}$, as these seem to be the two hypotheses of interest and ${H}_{c}$ is theoretically very unlikely.

We start by specifying the effect size, variance, and total sample size per study as computed by Lammertink et al. (2017; see https://osf.io/4exbz/).
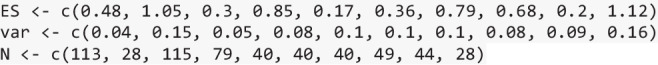


This is all the information we need to compute the study-specific PMPs with the *bain* package.^4^ Note that bain() always returns three different sets of PMPs: PMPa contains the PMPs of the hypotheses specified by the user, PMPb adds the unconstrained hypothesis ${H}_{u}$ to the set of specified hypotheses, and PMPc adds ${H}_{c}$, that is, the complement of the union of the hypotheses specified by the user. To test ${H}_{1}$ separately against both ${H}_{c}$ and ${H}_{u}$, we therefore only need to specify ${H}_{1}$ in the call to bain(), as PMPb will then test ${H}_{1}$ against ${H}_{u}$, and PMPc will test ${H}_{1}$ against ${H}_{c}$. In contrast, to test ${H}_{1}$ against ${H}_{0}$, we must specify both hypotheses; PMPa then contains the result we need. The code below computes the study-specific PMPs.
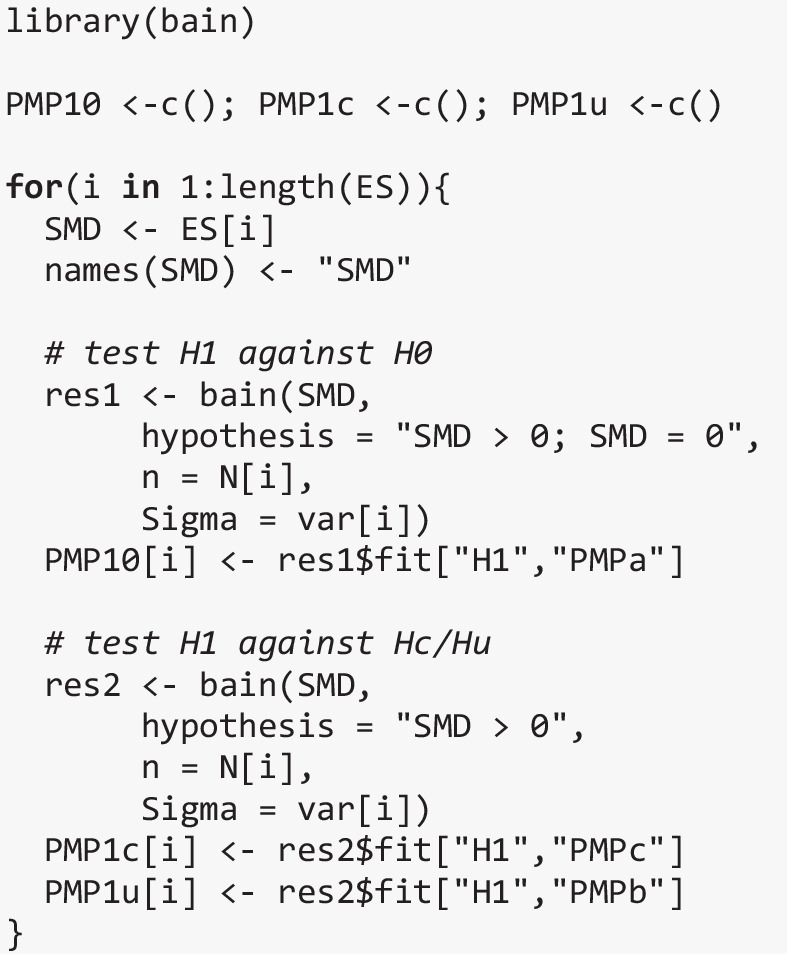


Now that we have computed the study-specific PMPs, we can compute the aggregated PMP for H1 against each of the alternatives with the code below (corresponding to Eq. 9).
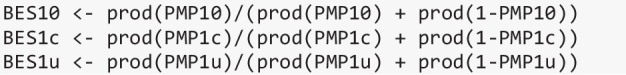


Table 1 shows the individual PMPs for ${H}_{1}$ for each study as well as the aggregated PMP. We do not show the (aggregated) PMPs for the alternative hypotheses, as these are simply 1-${PMP}_{1.}$. The results show that regardless of the considered alternative hypothesis, we obtain overwhelming evidence in favor of ${H}_{1}$, congruent with the result from the meta-analysis reported by Lammertink et al. (2017). Notably, we see that this is the case despite variation in the *study-specific* PMPs across the alternative hypotheses. Due to the relatively small sample sizes per study, ${H}_{0}$ receives more evidence than ${H}_{1}$ in those studies where the estimated effect size is small (note that each study only had 36–79% power to detect a medium effect). In contrast, ${H}_{c}$ never receives more evidence than ${H}_{1}$, since all effect sizes are positive and thus provide no evidence in favor of a negative effect size. Finally, ${H}_{1}$ receives more support than ${H}_{u}$ in each study, but the maximum study-specific PMP is .67, as this is the upper limit of ${PMP}_{1u}$ given the complexity of ${H}_{1}$ (see Introduction). Despite these differences in the study-specific PMPs, the aggregated evidence across all studies is clearly in favor of ${H}_{1}$ with all aggregated PMPs being greater than .99.

**Table 1:** Study-specific and aggregated posterior model probabilities for ${H}_{1}$ against each of the alternative hypotheses (${H}_{0}$, ${H}_{c}, {H}_{u}$) for the studies reported in the meta-analysis by Lammertink et al. (2017)

Study	PMP_10_	PMP_1c_	PMP_1u_
1. Evans et al. (2009)	.77	.99	.66
2. Evans et al. (2010)	.94	>.99	.67
3. Lukacs & Kemeny (2014)	.29	.91	.65
4. Mayor-Dubois et al. (2014)	.95	>.99	.67
5. Hsu et al. (2014)^a^	.20	.70	.58
6. Hsu et al. (2014)^b^	.35	.87	.64
7. Hsu et al. (2014)^c^	.88	.99	.67
8. Haebig et al. (2017)	.84	.99	.66
9. Grunow et al. (2006)	.22	.75	.60
10. Torkildsen (2010)	.95	>.99	.67
Aggregated PMP	> .99	>.99	> .99

*Note.* ^a^Low variability (X = 2) condition. ^b^Mid variability (X = 12) condition. ^c^Low variability (X = 24) condition

As mentioned above, it is also possible to test multiple hypotheses simultaneously. For example, if we were to test ${H}_{1}$, ${H}_{0}$, and ${H}_{u}$ simultaneously, we would run the following code to compute the study-specific PMPs.
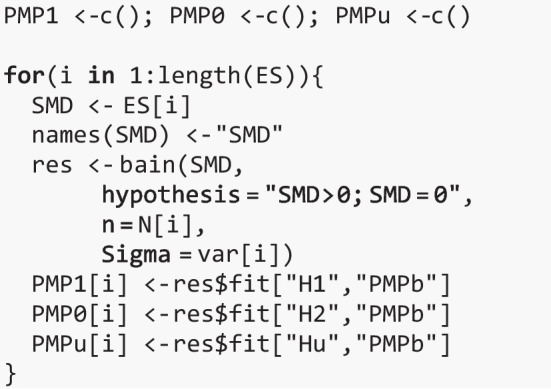


The aggregated PMPs are then computed according to Eq. (8). In the code below, BES1 gives the evidence in favor of ${H}_{1}$ relative to both ${H}_{0}$ and ${H}_{u}$, BES2 gives the evidence for ${H}_{0}$ relative to both ${H}_{1}$ and ${H}_{u}$, and BESu gives the evidence for ${H}_{u}$ relative to both ${H}_{1}$ and ${H}_{0}$.
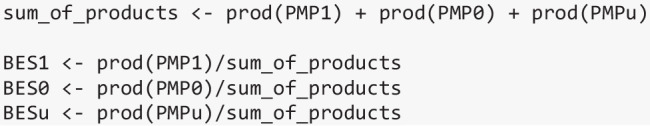


The results show that also when we test ${H}_{1}$, ${H}_{0}$ and ${H}_{u}$ simultaneously, ${H}_{1}$ (aggregated PMP = .995) clearly receives more support than both the null hypothesis (aggregated PMP < .001) and the unconstrained hypothesis (aggregated PMP = .005).

Finally, to illustrate that different types of hypotheses can be formulated and tested, we now show with the code below how to evaluate a range-constrained hypothesis (i.e., ${H}_{1}$: 0.5 < SMD < 0.8) against its complement (not ${H}_{1}$). ${H}_{1}$ now tests a more specific hypothesis than before: Whereas before we only tested whether DLD had a negative effect on auditory verbal statistical learning, we now test whether this is a *medium* negative effect.
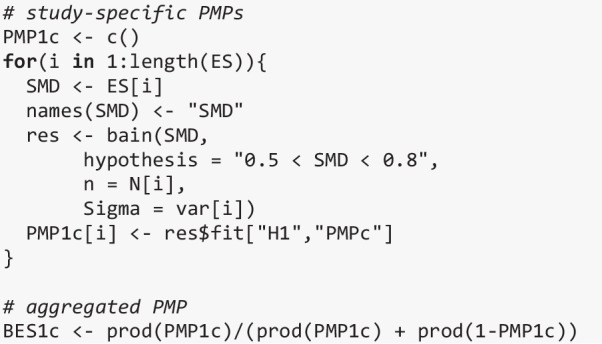


In line with the conclusion from the meta-analysis, there is compelling evidence that there is a medium negative effect of DLD on statistical learning when this hypothesis is tested against its complement (aggregated PMP > .99).

To facilitate the use of BES for new users, we have converted the code in this empirical demonstration into a wrapper function called bes(), which can be downloaded from our OSF page (https://osf.io/gbtyk/). Note, however, that this is not a general function for BES, as it can only handle single-parameter hypotheses; it uses *bain* and *bain*’s default priors for the parameter estimates to compute the study-specific PMPs; and it assumes equal prior model probabilities.

## Discussion

In the current study we introduced Bayesian evidence synthesis as a flexible alternative to meta-analysis for situations in which meta-analysis is difficult or impossible. When the set of studies a researcher wishes to combine is heterogeneous in terms of research design, participant characteristics or operationalization of key variables, it may not be possible to combine these studies by means of meta-analysis. In these situations, BES can be applied to investigate which hypothesis receives the most aggregated support across studies. As explained in the Introduction, the main advantage of BES is that it poses less constraints on the studies to be combined, as support for each hypothesis is first estimated in each study separately. This means that as long as each study provides independent evidence for the same overarching theory, BES allows for differences in the parameter estimates across studies and for study-specific hypotheses that include design and data characteristics unique to that study. Additional benefits of BES are that it allows for (i) testing multiple hypotheses simultaneously, and (ii) formulating and testing informative hypotheses that, unlike the conventional null hypothesis (${H}_{0}$: no effect) and its complement (not ${H}_{0}$), can directly test a specific theory or expectation. BES comes with the disadvantage, however, that unlike meta-analysis, it is only concerned with hypothesis testing and therefore does not allow for quantifying the effect size or the level of heterogeneity among effect sizes across studies. In addition, given that BES is still a relatively novel technique, no methods currently exist within the context of BES to deal with dependent effect sizes, assess and correct for publication bias, or test study-level predictors of degree of evidence across studies (analogous to meta-regression). Further developing BES is part of our research agenda, so future developments of the method may address some of these issues.

The goal of our simulation study was to compare the performance of BES and meta-analysis to illuminate under which conditions the two methods behave similarly and under which conditions their results diverge. Results were expected to sometimes differ, given that BES and meta-analysis answer a slightly different synthesis question: Whereas meta-analysis indicates whether the target hypothesis is supported by the pooled data, BES indicates the hypothesis that best describes each study. The results showed that in most scenarios, BES behaves similar to meta-analysis, in that the acceptance rate of the correct hypothesis increases with larger sample sizes and more studies. The two main exceptions were (i) when all individual studies were underpowered and (ii) when the true parameter value was on the boundary of the tested hypotheses. We will now discuss both situations in turn.

When all individual studies are underpowered, testing against the null or the unconstrained hypothesis can be problematic. As underpowered studies each provide more evidence for the null hypothesis than for the hypothesis of interest, BES only aggravates this issue as combining more studies will then increase our confidence that the null hypothesis best describes each individual study. Similarly, we saw that if studies are underpowered and there is large variation in study-specific parameter values, the unconstrained hypothesis will typically receive more evidence than the hypothesis of interest as the unconstrained hypothesis always describes the data well, whereas the hypothesis of interest will then provide a poor fit to at least some of the studies. This is an important limitation of BES, as underpowered studies are prevalent in the field of experimental psychology and one of the goals of synthesizing multiple studies may therefore be to reduce power issues. On the other hand, we found that including one underpowered study hardly impacted the results, even when combining as few as three studies. This suggests that power issues are mainly a problem when all or most studies are underpowered, but future studies should further investigate this.

When testing a target hypothesis against its complement when the true parameter value is on the boundary of these hypotheses, BES can yield strong support for either hypothesis (in the simulation, the target hypothesis was accepted in approximately 50% of the simulation replications). This issue extends beyond BES, as strong support for either hypothesis can already be found within a single study. However, BES does not help in solving this issue. Three potential solutions are discussed by Volker (2022), namely testing against an equality-constraint hypothesis (e.g., ${H}_{0}$) instead of or in addition to the complement, evaluating a hypothesis with a boundary on the minimum relevant effect size, or testing against both the complement and the unconstrained hypothesis in turn and see if both render support for (or against) the target hypothesis. We are currently still investigating which solutions work best in this scenario.

An additional finding was that testing against the unconstrained hypothesis was the most sensitive to the cut-off value used for accepting the hypothesis of interest (cf. Figs. 1, 2, 3 and 4 to Figs. S1-S4 on https://osf.io/gbtyk/). Because the PMP testing a hypothesis ${H}_{i}$ against the unconstrained hypothesis ${H}_{u}$ within a single study has an upper limit that is determined by the complexity of ${H}_{i}$, multiple studies will need to be combined to reach an *aggregated* PMP that is above this upper limit, even when all studies provide a perfect fit for ${H}_{i}$. This is not the case when testing against the null or complement hypothesis, as these PMPs do not have an upper limit. This once again shows the importance of considering both the fit and complexity of the hypotheses under consideration when interpreting the results. It also shows that it may be inappropriate to use the same cut-off values across different (sets of) hypotheses to decide what constitutes “strong” evidence for a hypothesis.

Regarding this last point, it is also important to note that using any kind of cut-off value for the aggregated PMP may give way to publication bias and questionable research practices in the same way as has been described for *p* values (e.g., Ioannidis, 2005; John et al., 2012; Simmons et al., 2011). In this study, we only used a cut-off value so that we could compute a common performance measure for meta-analysis and BES. However, when conducting BES, using a cut-off value is not strictly necessary because PMPs are interpretable by themselves. For a single study, a PMP of .90 means there is a conditional error probability of 10% that one of the other considered hypotheses is more appropriate (Hoijtink et al., 2019). The interpretation of an *aggregated* PMP is a bit more complicated, however, as different scenarios can result in the same aggregated PMP. For instance, when half of the studies support one hypothesis and half of the studies support the alternative hypothesis to the same degree (e.g., with study-specific PMPs of .90 vs .10), this leads to an aggregated PMP of .50 for both hypotheses. However, if all study-specific PMPs are .50 for both hypotheses, this also leads to an aggregated PMP of .50. In both cases we would conclude that neither hypothesis is a good description of all studies, but in the first case this is because the support for the hypotheses varies across studies, whereas in the second scenario there is no strong support for either hypothesis in *any* study. For this reason, it is important to always interpret the aggregated PMP in relation to the study-specific PMPs (cf. Kevenaar et al., 2021). If the study-specific PMPs show large variation, researchers may then try to explain this variation based on study characteristics. Of course, researchers may already expect variation in study-specific PMPs based on certain study characteristics *a priori* and may therefore not be very interested in the global support for a hypothesis. In that case, researchers could opt to perform multiple Bayesian evidence syntheses on specific subsets of studies.

Finally, we would like to point out a few limitations of the current study and provide some suggestions for future research. Because we wanted to show when BES results diverge from what researchers may expect based on meta-analysis, we focused on simple situations in which both methods are feasible. Therefore, we only considered single-parameter hypotheses and evaluated only two hypotheses at a time, and we did not vary data characteristics across studies such as different operationalizations of key variables. However, some of these factors were investigated by Volker (2022), who showed how the performance of BES varies as a function of the complexity of the study-specific hypotheses by varying the operationalization of key variables across studies. Volker also investigated how BES behaves for single- versus multiple-parameter hypotheses and when hypotheses are only partially correct (rather than either correct or incorrect). A second limitation is that we only looked at the acceptance rate as a performance measure. We did this to have a comparable performance measure for both BES and meta-analysis, but for BES it is more insightful to look at the value of the aggregated PMP directly, as this may show more nuanced differences than what we were able to show here. However, we refer the reader to Klugkist and Volker (2023) and Volker (2022) for partially similar simulation conditions where the authors did report the value of the aggregated BFs/PMPs. Future research could test how the number of considered hypotheses affects the BES results and develop guidelines for interpreting the value of the aggregated PMP that take the complexity of the tested hypotheses into account. Moreover, BES would greatly benefit from methods that allow for testing study-level predictors of degree of support and from methods that can handle dependent effect sizes. Finally, future studies may further explore possible solutions to deal with underpowered studies in the context of BES.

## Recommendations

Based on this study, we provide the following recommendations for potential users of BES:

- BES can be considered as a possible alternative to meta-analysis if model estimates across studies cannot be converted into a comparable measure, or if heterogeneity in the study designs or samples calls into question whether the effect sizes test the same underlying true effect, and, therefore, whether these effect sizes can be meaningfully aggregated or compared. Secondly, BES can be considered if the researcher wishes to evaluate an informative hypothesis directly (rather than only rejecting the null hypothesis or not), or if there are more than two competing hypotheses and the researcher wishes to evaluate all relevant hypotheses simultaneously.
- As BES provides joint support for a hypothesis relative to the other hypotheses considered, it is important to include all plausible hypotheses. It is also generally recommended to either include the unconstrained or the complement hypothesis to avoid choosing between hypotheses that do not represent the data well (i.e., the best hypothesis out of a set of bad hypotheses is still a bad hypothesis).
- When (some of) the studies are underpowered, it is important to be aware that BES is not a data-pooling approach and thus, does not increase power by aggregating studies. When equality-constrained hypotheses (e.g., ${H}_{0}$) are deemed sufficiently unlikely, one can evaluate the informative hypothesis of interest against its complement; the comparison that is least affected by low power.
- When interpreting the strength of support for a hypothesis, it is important to acknowledge that the complexity of the hypotheses under consideration impact the degree of (study-specific) support for each hypothesis, as well as the number of studies needed to achieve a certain level of aggregated support (see Volker, 2022). More generally, it is advised to always examine and report the individual study results in addition to the aggregated results from BES.
- If researchers are primarily interested in study-level factors that affect the support for a given hypothesis, then there are currently two options: (i) decide *a priori* which study-level factors are expected to impact the support for the hypotheses of interest and then perform BES on specific subgroups of studies, or (ii) try to detect patterns post hoc by carefully examining the study-specific PMPs.

On a final note, we would like to stress once more that BES is a relatively novel technique that is still under development. Some of the limitations mentioned in this paper may therefore be addressed by future developments of the method. Likewise, as we further investigate the behavior of BES under different circumstances, the current recommendations may be adjusted.

## Footnotes

1 As explained in Hoijtink et al. (2019, Footnote 1) this is “because, loosely spoken, among the infinite number of possible combinations of values for μ_1_, μ_2_, and μ_3_ that are in agreement with H_u_, μ_1_ = μ_2_ = μ_3_ has a ‘zero probability’ of occurring.” So whether μ_1_ = μ_2_ = μ_3_ is included in the hypothesis (as in H_u_) or not (as in H_c_) will not affect the Bayes factor.

2 Alternatively, we could have iteratively resampled a new set of studies for each value of *k*. Note, however, that since we conducted 1000 replications per simulation condition, the mean and variance of the observed effect sizes very closely reflect the values we specified when generating the data. The results would therefore be virtually identical if we had resampled a new set of studies for each value of *k*. Here, we chose to cumulatively add the *K* studies because this nicely shows what happens if new studies are added to the existing set of studies. This is fitting in the context of research synthesis since all systematic reviews, including meta-analyses, are advised to be updated (more or less) regularly as new evidence becomes available (Garner et al., 2016).

3 Although it is uncommon to see meta-analytic papers with only five studies, many meta-analytic papers report on separate meta-analyses for different outcome measures (e.g., the 747 meta-analyses collected by van Erp et al. were published in only 61 papers). A set of five studies per meta-analytic outcome is not uncommon, as illustrated by the fact that 25% of the meta-analyses reported by van Erp et al. synthesized five studies or less.

4 Note that for multiple-parameter hypotheses (i.e., hypotheses that involve more than one effect, such as two main effects and an interaction), *bain* requires the variance–covariance matrix of the parameter estimates rather than just the variance of each estimate.

## Open Practices Statement

In accordance with the Peer Reviewers' Openness Initiative (https://opennessinitiative.org, Morey et al., 2016), all simulated data, code, and supplementary figures associated with this manuscript were available during the review process and remain available on OSF project “Bayesian evidence synthesis” at https://osf.io/gbtyk/. None of the analyses were preregistered.
